# Infectious Diseases in Internationally Adopted Children and Intercountry Discrepancies Among Screening Protocols, A Narrative Review

**DOI:** 10.3389/fped.2019.00448

**Published:** 2019-11-07

**Authors:** Elena Chiappini, Barbara Bortone, Sara Borgi, Sara Sollai, Tommaso Matucci, Luisa Galli, Maurizio de Martino

**Affiliations:** Department of Health Science, Anna Meyer Children University Hospital, University of Florence, Florence, Italy

**Keywords:** internationally adopted children, infectious diseases, screening, protocols, tuberculosis, parasites

## Abstract

Internationally adopted children (IAC) require thorough health assessments at time of arrival in the host country. As these children are at higher risk for infectious diseases, such as gastrointestinal parasites, tuberculosis, hepatitis, syphilis, and human immunodeficiency virus, early diagnosis of infectious diseases is fundamental for the optimal management of the child and, also, to reduce the risk of transmission to the adopting community. Comparative analysis of the screening protocols adopted in Europe, the United States, and Canada revealed different approaches to the adopted children. A homogeneous and internationally shared standard of care in the management of IAC should be provided.

## Introduction

Infectious diseases are commonly reported in internationally adopted children (IAC) (1), and their need for rapid, cautious, and thorough screenings has been underlined by several authors (1–5). A recent Italian study indicated that clinical conditions affecting IAC, ranging from congenital malformations, to complex infectious diseases, such as tuberculosis infection (observed in 15% of children included) and parasitosis (in more than 20%), to non-severe and easily treatable infections, such as *Molluscum contagiosum* and fungal skin infections, have been frequently reported. Notably, about 40% of IAC presented at least one infectious disease (2).

Although IAC require medical evaluation and health certifications before adoption, many diseases may remain undiagnosed (3–5).

Early diagnosis of infectious diseases is crucial both for the optimal management of IAC and to reduce risk of transmission to the community (1, 6, 7). Cases of transmission of the Hepatitis A and Hepatitis B viruses, scabies, tuberculosis, *Tinea capitis*/*corporis*, and measles to adoptive families have been reported in the literature (1, 6, 7).

A narrative review of the literature was conducted to identify and compare the screening protocols used by different countries and to discuss the major discrepancies between them.

## Methods

Post-adoption screening protocols were identified by searching the guidelines websites and national adoption association websites of countries reported to receive the highest number of IAC (United States, Italy, Spain, Canada, and France) (8). Moreover, PubMed was consulted with the search strategy: (international^*^OR abroad) AND (“adopted children” OR adoption OR adoptees) AND (“infectious diseases” OR infection^*^). The reference lists of the retrieved articles were analyzed. A comparison among the retrieved protocols was performed, focusing on screening for infectious diseases. Moreover, a narrative review of the literature was performed to discuss discrepancies.

## Results

Six different protocols were identified (9–14), published in the United Kingdom (UK), Spain, Italy, Canada, France, and the United States (US). Four protocols were retrieved by searching national adoption association websites (10–12, 14) and two by searching the reference lists of results from PubMed (9–13). Except for the French protocol, which is provided by the Centre Hospitalier Universitaire de Nantes (13), all the others were national protocols (9–12, 14). The English protocol was published in 2004 (9), the Spanish protocol in 2008 (10), and the four others between 2013 and 2017 (11–14). Their recommendations have been summarized in Table 1.

**Table 1:** Laboratory microbiological tests recommended in different screening protocols.

**Laboratory tests**	**UK 2004 (9)**	**Spain 2008 (10)**	**Italy 2013 (11)**	**Canada 2014 (12)**	**France 2015 (13)**	**US 2017 (14)**
HBV serologic testing	To all IAC; repeat after 3 months; HbSAb, HBSAg, anti-HBc Ab	To all IAC; HbSAb, HBSAg, anti-HBc Ab	To all IAC; consider window period Antibodies not specified.	To all IAC; repeat after 6 months HbSAb, HBSAg	To all IAC HbSAb, HBSAg, anti-HBc Ab	To all IAC; repeat after 6 months HbSAb, HBSAg
HCV antibody	To all IAC; repeat after 3 months in IAC at risk	To all IAC; repeat after 6 months if clinical suspicion	To all IAC; consider window period	To all IAC; repeat after 6 months	To all IAC	To all IAC; repeat after 6 months
HAV serologic testing (IgG; IgM)	Not recommended	To IAC coming from Latin America	To all IAC	To all IAC	To all IAC	To all IAC
HIV 1-2 serologic testing	To all IAC; repeat after 3 months in IAC at risk	To all IAC; repeat after 3–6 months in IAC at risk	To all IAC; consider window period	To all IAC; repeat after 6 months	To all IAC	To all IAC
Non-treponemal tests	To all IAC	To all IAC	Not specified	To all IAC	To all IAC	To all IAC
Treponemal tests	Not recommended	If non-treponemal test is positive	Not specified	Not recommended	To all IAC	To all IAC
TST	To all IAC; repeat after 6 months if negative	To all IAC	To all IAC	To all IAC; or IGRA	To all IAC + chest x-ray	To all IAC; preferred <5 years of age; repeat after 6 months from arrival; or IGRA
IGRA	Not recommended	Not recommended	If TST is positive	To IAC older than 2 years; or TST	Not recommended	To all IAC; preferred ≥5 years; repeat after 6 months from arrival; or TST
Ova and parasite exam	Only to IAC at risk	To all IAC	To all IAC	To all IAC	To all IAC	To all IAC
*Giardia lamblia* antigen	Not recommended	Not recommended	Not recommended	Not recommended	Not recommended	To all IAC; 1 sample
*Cryptosporidium* spp. antigen	Not recommended	Not recommended in	Not recommended	Not recommended	Not recommended	To all IAC; 1 sample
*Toxocara canis/cati* specific antibodies	Not recommended	Not recommended	Hypereosinophilia with negative parasitic stool examination	Not recommended	Not recommended	Hypereosinophilia with negative parasitic stool examination
*Strongyloides* spp. specific antibodies	Not recommended	Not recommended	Hypereosinophilia with negative parasitic stool examination	To IAC from endemic areas	Not recommended	Hypereosinophilia with negative parasitic stool examination
*Schistosoma* spp. specific antibodies	Not recommended	Not recommended	Hypereosinophilia with negative parasitic stool examination	To IAC from endemic areas	Hypereosinophilia	To IAC from endemic areas
*Trypanosoma cruzi antibodies*	Not recommended	IAC from Latin America	Not recommended	Not recommended	Not recommended	IAC from Mexico, Central America, and South America
Stool culture	Clinical suspicion	Clinical suspicion	Clinical suspicion	Clinical suspicion	Clinical suspicion	Clinical suspicion
Tests for *Malaria*	To all IAC from an endemic area; repeated if clinical suspicion	Clinical suspicion in IAC from India, Asia, Sub-Saharan Africa, Latin America	Clinical suspicion	Clinical suspicion	Clinical suspicion	Clinical suspicion in IAC from an endemic area

*HBV, Hepatitis B virus; HCV, Hepatitis C virus; HAV, Hepatitis A virus; HIV, Human Immunodeficiency Virus; TST, tuberculin skin test; IGRA, Interferon Gamma Release Assay*.

## History

All the retrieved protocols include a proper analysis of history to describe the adoption process, the child's life before adoption, any host institutions where the child lived, the child's psychophysical development, and any certified vaccinations. Recent and remote pathological anamnesis, previous laboratory tests, instrumental examinations, and specialist evaluations, whenever available, are necessary. Additionally, clinical histories of the biological family need to be reviewed and added to the report (9–14).

## Skin Problems

A global clinical evaluation should be performed with particular attention to skin and genital examination (9–14).

Skin infections in IAC are very common (6); having lived in an overcrowded institution and coming from a tropical country are the main risk factors for this type of infection. The most frequently reported dermatological disease is *Tinea capitis* infection, followed by *Molluscum contagiosum*, pediculosis of the head, and impetigo; scabies is frequently observed and should be promptly diagnosed and treated (6, 7). Skin examination is also crucial to detect a Bacille Calmette Guerin (BCG) scar and scars compatible with previous infections, such as varicella zoster infection (9–14).

## Screening Tests

### Tuberculosis

While the tuberculin skin test (TST) is the first choice in the UK, Spain, Italy, and France (9–11, 13), Interferon-γ release assays (IGRA) can be an acceptable screening alternative in Canada and the US in children older than 5 and 2 years, respectively (12, 14).

### HAV

Screening for hepatitis A (HAV) is not routinely recommended in IAC in UK and Italy (9, 11), but it is suggested in Canada France, and the US (12–14) and in IAC from Latin America arriving in Spain (10).

### HBV

HBV screening is recommended by all of the guidelines reviewed. Italy, Canada, and the US recommend HBsAg and HBsAb (11, 12, 14), whereas the UK, Spain, and France also include HBcAb (9, 10, 13). The UK, Italy, Canada, and the US recommend repeating the serology after a window period if the first test is negative (9, 11, 12, 14).

### Malaria

Only the UK protocol recommends routine screening for malaria upon arrival in all IAC coming from an endemic area (9), while all the other protocols suggest it only in IAC coming from endemic areas with fever (13) and/or hepato-splenomegaly (10–12, 14).

### Other Parasitoses

An ova and parasite exam on three stool samples is routinely recommended by all of the protocols, except for that in the UK, which recommends the test only to children who have lived in poor sanitary conditions, have been institutionalized, or have an anamnestic history of diarrhea (9). Serologic testing for *Strongyloides* spp. and *Toxocara canis* is recommended in children where there is clinical suspicion or with eosinophilia and a negative ova and parasite exam in Italy and the US (11, 14); only in Canada is *Strongyloides* spp. routinely investigated in all IAC coming from Africa and Southeast Asia (12).

Serologic testing for Schistosoma is indicated in Canada and the US in IAC coming from endemic areas (12, 14).

Serologic testing for *Trypanosoma cruzi* is routinely performed only in Spain and in US IAC (10, 14) coming from endemic countries, while in other countries this test is not recommended (9, 11–13).

Anti-*cysticercus* IgG antibodies are not recommended by any protocol, not even among IAC coming from endemic countries (9–14).

### Syphilis

All the retrieved protocols recommend screening for syphilis, but there is no concordance as regards the choice of treponemal or nor-treponemal antibodies. Only non-treponemal serologic testing is recommended in the UK, Spain, and Canada (9, 10, 12); both treponemal and non-treponemal tests are routinely indicated in France and in the US (13, 14). The Italian protocol does not specify the serologic pattern required (11).

## Discussion

This paper focuses on the screening protocols for infectious diseases in newly arrived IAC. It is important to specify that many other issues must be considered when managing IAC, such as immunization coverage, auxo-endocrinological problems, and other non-infectious diseases.

To the best of our knowledge, this is the first study comparing existing protocols for the screening of infectious diseases in IAC. A limitation of this study is that some protocols may have been missed by our research and that the comparison among the protocols may be affected by the different levels of methodological quality and different years of publication. Moreover, these protocols might not reflect the common practice in these countries, since heterogeneity has been reported among different centers in the same country, and more recent evidence might guide current management (2).

Most protocols were provided on a national level, whereas in France, a systematic national approach does not exist, and we reported the regional approach of a Medical Guidance for Adopted Children Consultation (13).

All the protocols report sets of screening tests that allow early detection of serious infectious conditions and those most commonly reported among IAC (9–14).

All the retrieved protocols recommend screening for human immunodeficiency virus (HIV), Hepatitis B virus (HBV), Hepatitis C virus (HCV), Tuberculosis, syphilis, and intestinal parasites (9–14).

The main discrepancies among the protocols regard the recommendations for screening of the Hepatitis A virus (HAV), malaria, and other parasitic infections.

HAV serology testing is not required by the UK protocol and is recommended only in children coming from an endemic area by Spanish protocol, whereas it is routinely recommended for all IAC by the other protocols. HAV infection among IAC is frequent, and cases of transmission to adoptive families have been reported (15–19). In an American study including 656 IAC, 4.6% were positive for HAV infection, even if they did not show any symptoms (15). They came mostly from Ethiopia and Guatemala (15). This screening may be important so that preventive measures can be started promptly, since many cases of HAV have been reported among the components of adoptive families (15–19).

Among the retrieved protocols, only the UK protocol recommends screening for malaria in IAC coming from endemic areas (9). The introduction of a screening test for malaria in IAC coming from malaria-endemic countries has also recently been supported in Italy after several cases were reported of asymptomatic children with malaria coming from the African continent (20). Either Polymerase Chain Reaction (PCR) or thin and thick strip microscopy have been reported as valid screening tests (20, 21).

Intestinal parasitic infections are very common in IAC: up to 27% of children arriving in the US may have one or more parasitic infections (1, 18, 22). In Italy, the prevalence of intestinal parasitic infections among IAC is 23% (3). The most frequently observed parasitic infections are *Giardia lamblia* and *Toxocara canis* (1, 18, 22). All the protocols routinely recommend an ova and parasite exam on three stool samples, except for the British one, which indicates this exam only whether a clinical suspicion is posed.

Only some of the protocols recommend serology testing for *Strongyloides* spp. and *Schistosoma* spp. where there is clinical suspicion or when the child comes from an endemic area. The recently published European Centre for Disease Prevention and Control (ECDC) protocol for screening of infectious diseases in migrants recommends screening of *Strongyloides* spp. and *Schistosoma* spp. among all migrants coming from endemic areas (23).

Other relevant differences among the protocols regard the choice of the elective test for the screening of latent tuberculosis infection (LTBI) and syphilis and the specific antibodies and the timing of serology of HBV.

Since many IAC have been vaccinated with BCG and have undergone numerous TSTs, the TST results are difficult to interpret, and discrepancies between TST and IGRA have frequently been reported (24, 25). Higher rates of positive TST were observed in children recently vaccinated with BCG, while TST positivity rates were less frequent in those who had been vaccinated less recently (15 years before) (26).

The ECDC protocol for screening and vaccination for infectious diseases in newly arrived migrants (either adults or children) to European Union countries indicates either the use of TST or IGRA but does not specify the preferred test per age (23).

It has been reported that IGRA showed higher specificity overall than TST, but it may have lower sensitivity in children under 5 years of age (1, 3, 24). For this reason, the US protocol and the recent Italian guidelines for the diagnosis of tuberculosis in migrants prefer the use of TST for children <5 years of age (27). On the other hand, some experts suggest the use of only IGRA also in children as young as 2 years if a good follow-up can be assured (28). Moreover, IGRA has been reported to be preferred when IAC are unlikely to return for TST reading (7, 8).

In a review of cost-effectiveness studies, LTBI screening in migrants was reported to be cost-effective according to seven studies, especially in young subjects from high-incidence countries. IGRA was the most cost-effective test for LTBI screening in migrants in four studies (29).

The prevalence of HBV infection among adopted children has been estimated to be around 2–5% (18, 30). It is essential to detect the infection in order to manage the infected child and to avoid contagion of adopting families.

Discrepancies have been found regarding the antibodies required and the timing.

During the window period, the only marker of acute infection is anti-HBc, which is highly specific for establishing the diagnosis of acute infection (31). For this reason, Spain recommends repeating the serology after 3–6 months if HBcAb has not been tested at arrival (10).

The recent ECDC protocol for screening of infectious diseases in migrants recommends dosing HBsAg, anti-HBc, and HBs-Ab to migrants from intermediate-/high-prevalence countries (≥2–≥5% HBsAg) (23).

The prevalence of syphilis amounted to 3% in two studies conducted on IAC (1, 18). The use of only one type of test may be insufficient for diagnosis because false-positive non-treponemal test results occur with various medical conditions and because treponemal test results remain positive long after syphilis has been treated adequately and can be falsely positive with other spirochetal diseases (32).

Many factors may justify these discrepancies. These protocols were published in different periods, with the British protocol being produced in 2004 and the Spanish in 2008 (9, 10). Therefore, new evidence may have been included in the protocols more recently produced by Italy, the US, France, and Canada (11–14).

Another relevant factor is that every receiving country deals with heterogeneous groups of IAC in terms of country of origin and age (1). This fact may affect the prevalence of infectious diseases in each group. For example, the serologic testing for *Strongyloides* spp. and *Schistosoma* spp. in IAC coming from endemic areas is routinely recommended by the Spanish protocol but not in the other countries. This may result from the high number of and greater experience with children coming from African countries (33).

Moreover, these features constantly change according to new adoptive policies in the countries of origin and the incidence of infectious diseases in that specific period (1). For these reasons, the frequency of infectious diseases among IAC is not easily measurable (1, 33).

Furthermore, the discrepancies between the screening protocols reflect the lack of international consensus regarding some areas, such as the appropriate test for LTBI (24, 25). Furthermore, different studies conducted in the same country report different screening protocols, showing that approaches are not only internationally heterogeneous (2).

Cost-effectiveness studies have been performed that documented the cost-effectiveness of some screening tests in migrants (either children or adults), but further studies may be needed focusing specifically on adopted children (22, 34). Recently, the ECDC has produced guidance on screening and vaccination for infectious diseases in newly arrived migrants within the European Union (23). This document is a useful instrument but has the limitation of dealing with both adults and children and does not focus specifically on IAC, who are a high-risk, unusual population.

A systematic approach categorized per country of origin and age may be useful, to be integrated with the information coming from the history and examination of the child.

Since the screening protocols used in different countries are not homogenous, the introduction of an optimized internationally shared protocol focusing on IAC would be highly desirable to fill the potential gaps in local protocols. Moreover, an international consensus on the proper screening of LTBI in this group of children would be highly desirable.

## Author Contributions

EC, BB, SB, SS, TM, MM, and LG conceived the study, reviewed the literature, and drafted the manuscript. All the authors read and approved the final manuscript.

### Conflict of Interest

The authors declare that the research was conducted in the absence of any commercial or financial relationships that could be construed as a potential conflict of interest.
